# Odontogenic Maxillary Sinusitis: A Case Report of Interdisciplinary Care in the Context of an ORL Emergency

**DOI:** 10.1155/crid/8875346

**Published:** 2026-02-12

**Authors:** Olivier Deny, Chiara Cecchin-Albertoni, Antoine Dubuc, Thibault Canceill

**Affiliations:** ^1^ RESTORE Research Center, Inserm/Université de Toulouse, Toulouse, France; ^2^ Service d′Odontologie-Médecine Bucco-Dentaire, Centre Hospitalier Universitaire de Toulouse, Toulouse, France, chu-toulouse.fr; ^3^ Département Odontologie, Faculté de Santé de Toulouse, Toulouse, France; ^4^ InCOMM (Intestine ClinicOmics Microbiota and Metabolism), Laboratoire i2MC UMR1297 Inserm/Université de Toulouse, Toulouse, France

**Keywords:** CT scan, maxillary sinusitis, ORL emergency, periapical infection

## Abstract

Odontogenic maxillary sinusitis (OMS) represents an important cause of unilateral sinus disease and can lead to persistent symptoms and repeated courses of antibiotics if unrecognized. Prompt identification of a dental source is essential to prevent chronicity and avoid unnecessary interventions. We report the case of a 40‐year‐old woman who presented with a 3‐week history of persistent right maxillary sinus pain, unresponsive to two antibiotic prescriptions. On examination, she had a buccal collection in the upper right maxilla, close to a previous Caldwell‐Luc surgical window. She did not present any signs of general condition deterioration. A contrast‐enhanced CT scan (single‐bolus iodinated contrast) demonstrated a complete opacification of the right maxillary sinus, leading to a diagnosis of obstructive maxillary sinusitis. A transfer into the eye–nose–throat (ORL) and cervicofacial surgery department was decided, in order to perform a middle meatal antrostomy. At the scheduled 2‐week ORL follow‐up, the patient reported gradual recurrence of symptoms, including pain and a sensation of heaviness in the right maxilla. Examination revealed persistent pus in the right maxillary sinus. As the presence of causal teeth was the most probable reason for this recurrence, the patient was admitted into the oral medicine department of the hospital for dental examination and radiography. A periapical infection, associated with root fracture, was revealed on Tooth #16 and treated with tooth extraction. The symptoms improved in the next following weeks. This case highlights the value of oral and craniofacial radiographies in a coordinated dental and ORL approach for the treatment of refractory maxillary sinusitis. Moreover, clinicians should maintain suspicion for odontogenic sources in unilateral sinusitis to optimize patient outcomes and reduce diagnosis errance.

## 1. Introduction

Maxillary sinusitis of odontogenic origin (OMS) is a recognized but frequently underdiagnosed clinical entity. It represents 5%–50% of all cases of maxillary sinusitis [1–5], depending on the populations and the geographical areas. Anatomically, the maxillary sinus maintains a close spatial relationship with the roots of the upper posterior teeth—particularly the first and second molars—rendering it highly susceptible to the spread of odontogenic infections such as periapical lesions, periodontal disease, or iatrogenic complications following dental procedures. This intimate anatomical connection allows bacteria from the teeth environment to breach the Schneiderian membrane and invade the sinus cavity, leading to both acute and chronic sinus manifestations [6]. Odontogenic maxillary sinusitis is thus unique in its dual pathophysiology, requiring both dental and otorhinolaryngological (ORL) expertise for effective management. Treatment strategies must address the source of infection—typically through root canal therapy or extraction—and often necessitate adjunctive sinus drainage or debridement. These medical and surgical procedures are especially proposed in persistent or complicated cases because, at first, endodontic management is considered as the first line of treatment [3, 5]. The need for ORL specialists may be essential, particularly when systemic health is compromised, symptoms are severe, or when conservative measures fail. Odontogenic sinusitis can manifest as an acute ORL emergency, presenting with facial edema, intense facial pain, purulent rhinorrhea, and even orbital complications, demanding rapid interdisciplinary intervention [7, 8]. Failure to recognize the dental etiology not only delays effective treatment but also increases the risk of complications such as chronic sinusitis, oroantral fistula, or secondary fungal and bacterial superinfections. Recently, Rolla et al. emphasized the need to address missed second mesiobuccal (MB2) canals in maxillary molars to ensure complete resolution of sinus involvement [9]. This reinforces the importance of advanced imaging techniques, including tridimensional ones, which can provide critical diagnostic detail and guide therapeutic decisions [10–12].

This case report is aimed at highlighting the pivotal role of interdisciplinary collaboration between dental and ORL professionals in the diagnosis and management of odontogenic sinusitis, especially in emergency settings where the origin of infection may be obscured by nonspecific sinonasal symptoms. By presenting a case in which initial surgical and medical treatment failed, we underscore the necessity of identifying and treating the underlying dental cause to achieve definitive resolution.

## 2. Case Presentation

A 40‐year‐old female with a history of depressive episodes and prior maxillary sinus surgery for aspergillosis in 2017 presented herself to the medical emergency department of Toulouse University Hospital (Toulouse, France), with a 3‐week history of persistent right maxillary sinus pain unresponsive to antibiotics (levofloxacin as a first‐line treatment for 10 days, then supplanted by amoxicillin/clavulanic acid association during 10 days as symptoms failed to improve). These prescriptions were delivered by two distinct ear–nose–throat specialists.

### 2.1. Initial Emergency Room Assessment and Management

On examination, the patient had a right hemifacial edema, persistent pulsatile, sleep‐disruptive pain, worsened by decubitus position, forward flexion of the head and food or beverage intake, reproducible with palpation, and a buccal collection in the upper right maxilla, close to a previous Caldwell‐Luc surgical window. Patient reported a fever episode the night before. She did not display any signs of systemic inflammatory response, as her C‐reactive protein concentration in blood was 0.6 mg/L and she had no leukocytosis. At the time of the consultation, she had a normal heart rate and blood pressure. She did not present any signs of general condition deterioration. Nasal endoscopy revealed a deviated septum but no secretions. After an ORL specialist recommendation, a computed tomography scan (CT scan, Toshiba Aquilion Prime, 112 mGy.cm) of the nasopharynx and sinus area with iodine contrast agent single‐bolus injection was performed.

### 2.2. CT Scan and Referring to ORL Department

The CT scan (Figure 1) did not reveal any abnormalities of the nasopharynx area, any suspect mass, or significant adenopathy. It did show a complete opacification of the right maxillary sinus, with a significant adjacent bony reaction. Evidence of prior right middle meatal antrostomy and anterior surgical approach was visible. In addition, there was an infiltration of premaxillary soft tissues, communicating with the sinus content through the previous surgical window, and periapical lysis of Teeth #16 and #17, with protrusion into the sinus. No opacification of the other paranasal sinuses or abnormalities of the orbital cavities were visible.

Figure 1CT scan of the sinus region. (a, b): Coronal views showing apex of the mesiobuccal (with periapical lesion, white arrow) and palatal roots of Tooth #16. There is a unilateral opacification of the right maxillary sinus with no evidence of drainage, in contrast to the normally aerated contralateral side (white stars). (c): Axial view revealing the previous surgical window (striped white arrow). (d): Sagittal view of the right maxillary sinus and apex of buccal roots of Tooth #16 (striped white arrow).

**Figure:**
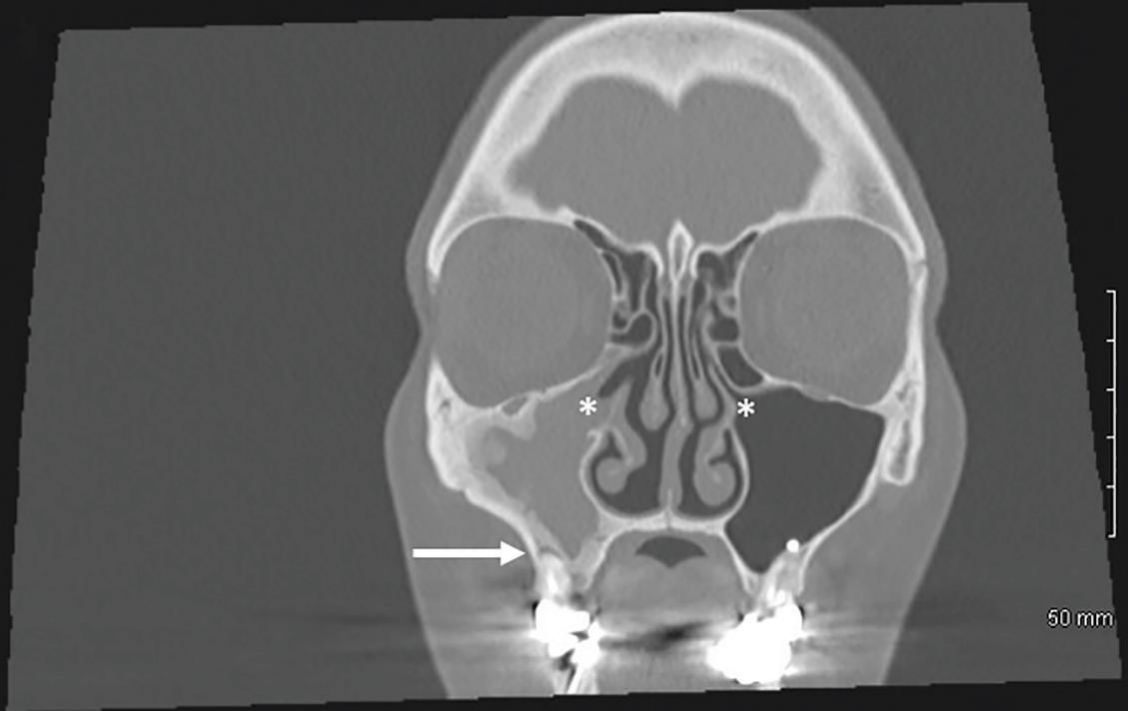
(a)

**Figure:**
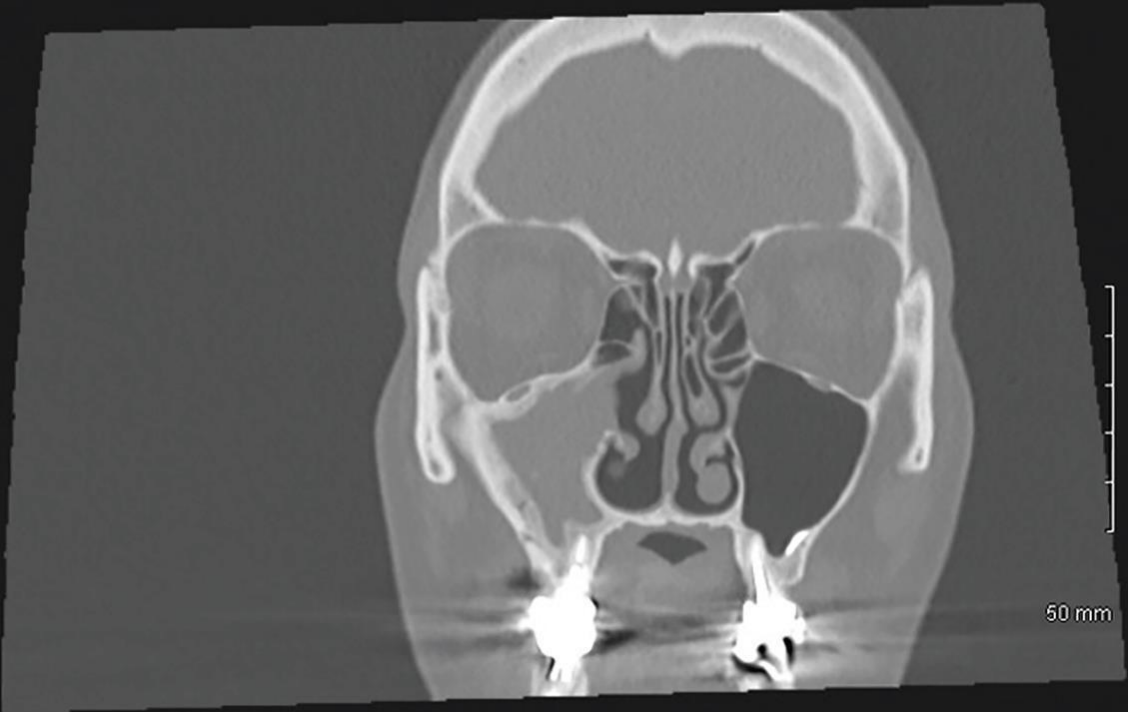
(b)

**Figure:**
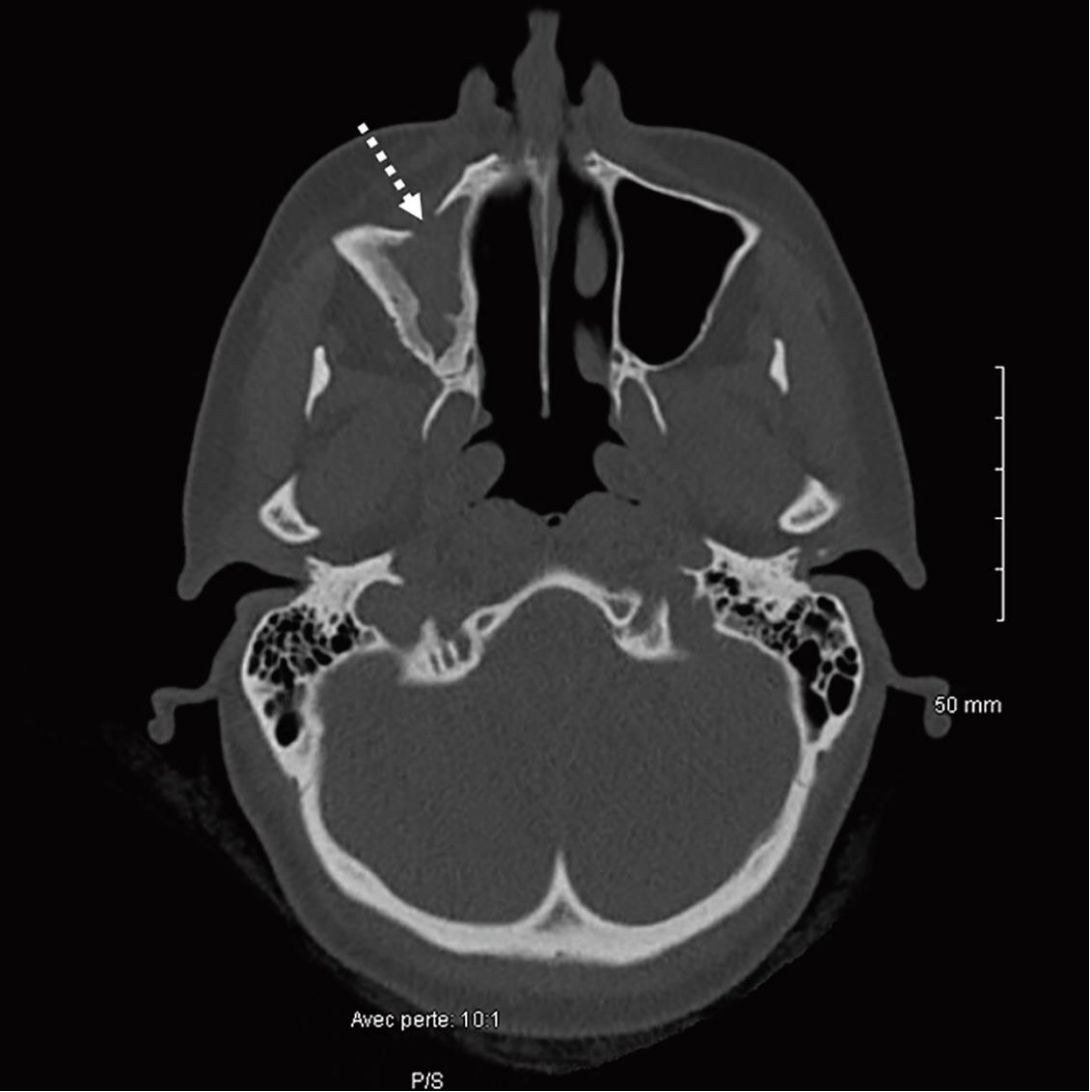
(c)

**Figure:**
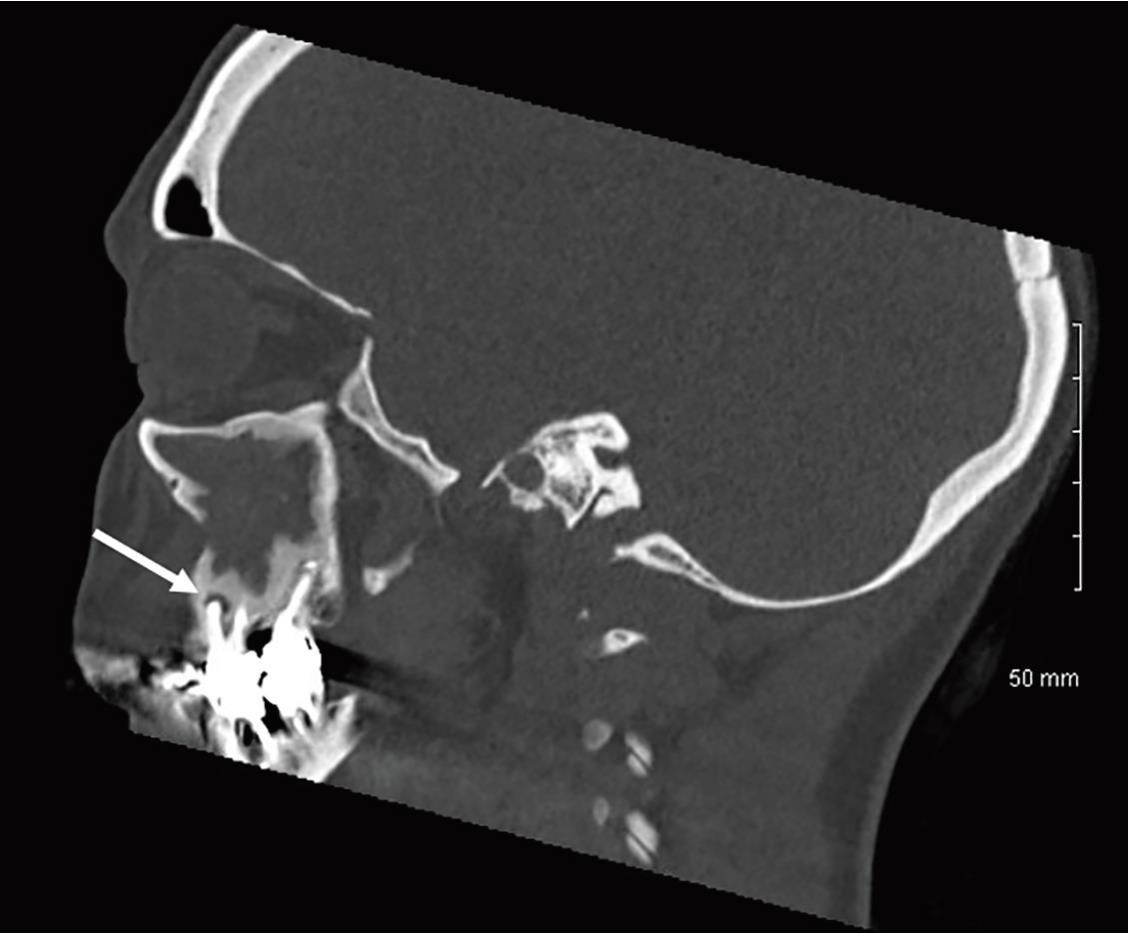
(d)

Following this examination, a diagnosis of obstructed right maxillary sinusitis was made, and a transfer into the eye–nose–throat and cervicofacial surgery department was decided, in order to perform a middle meatal antrostomy.

### 2.3. ORL Surgery Management

The patient was admitted and underwent middle meatal antrostomy and biopsy under general anesthesia. Postoperatively, the patient received broad‐spectrum intravenous antibiotics (piperacillin–tazobactam, Tazocilline 2 g/250 mg) at a rate of two perfusion bags every 8 h to cover common Gram‐positive and Gram‐negative pathogens [13]; this was continued for 48 h. Multiple biopsy sampling, performed at the time of the surgery, later revealed inflammatory tissue with fungal mycelia consistent with aspergillosis (Grocott and PAS positive), without any evidence of malignancy. The patient′s symptoms improved rapidly, and she was discharged 2 days later, with instructions to seek dental evaluation. Initial intravenous antibiotics were supplanted by oral administration of the association of amoxicillin and clavulanic acid (Augmentin 1 g/125 mg, one tablet three times a day during 7 days) in regard to the clinical improvements at the time of discharge. Local care consisted of nasal cavities rinsing with saline solution three times a day for a month and paracetamol 1 g up to four times a day in the event of pain. A follow‐up appointment has been set up 2 weeks after the discharge.

### 2.4. Relapse and Dental Referral

At the scheduled 2‐week ORL follow‐up, the patient reported a gradual recurrence of symptoms, including pain and a sensation of heaviness in the right maxilla. Examination revealed an open meatotomy but persistent pus in the right maxillary sinus. Given the high likelihood of an odontogenic source for the relapse, the patient was referred to the oral medicine department of Toulouse University Hospital (Toulouse, France) for dental assessment and radiography. Dental examination revealed a fixed dental bridge consisting of multiple crowns (Teeth #13, #15, #16, and #17) and one pontic in place of Tooth #14. No pulp vitality testing was performed as these four teeth presented root canal treatments that appeared satisfactory on 2D radiographs (Figure 2). However, axial percussion on Tooth #16 elicited pain, and a buccal edematous fistula, bloody on contact with the examination mirror, was visible adjacent to Teeth #15 and #16. Sectorial dental X‐rays showed a periapical lesion on the mesio‐vestibular root of Tooth #16, a post in the palatal root, and a possible root fracture (Figure 2b).

Figure 2(a): Intraoral view of the upper right quadrant revealing a buccal fistula. (b): 2D radiograph of the posterior teeth.

**Figure:**
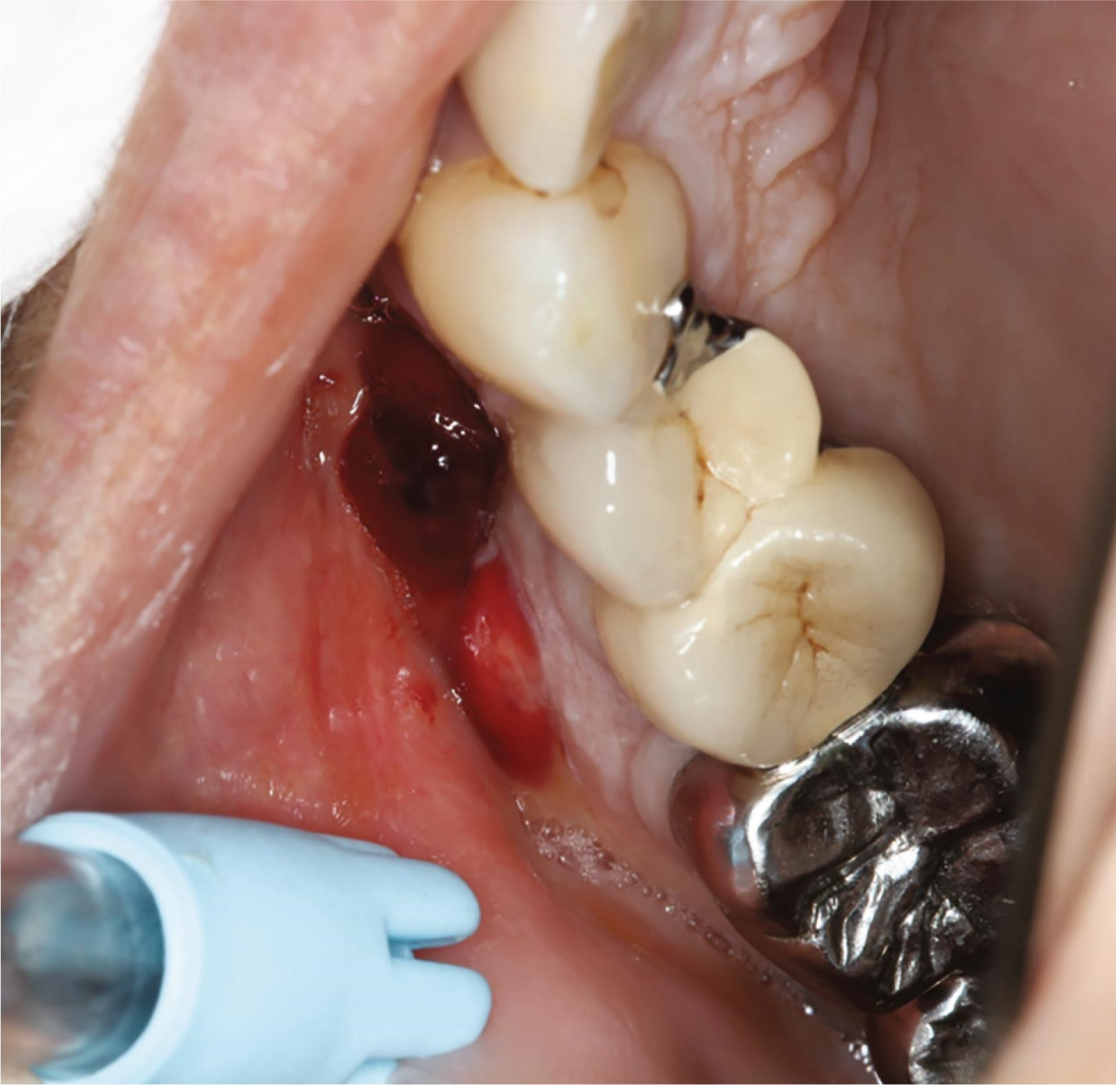
(a)

**Figure:**
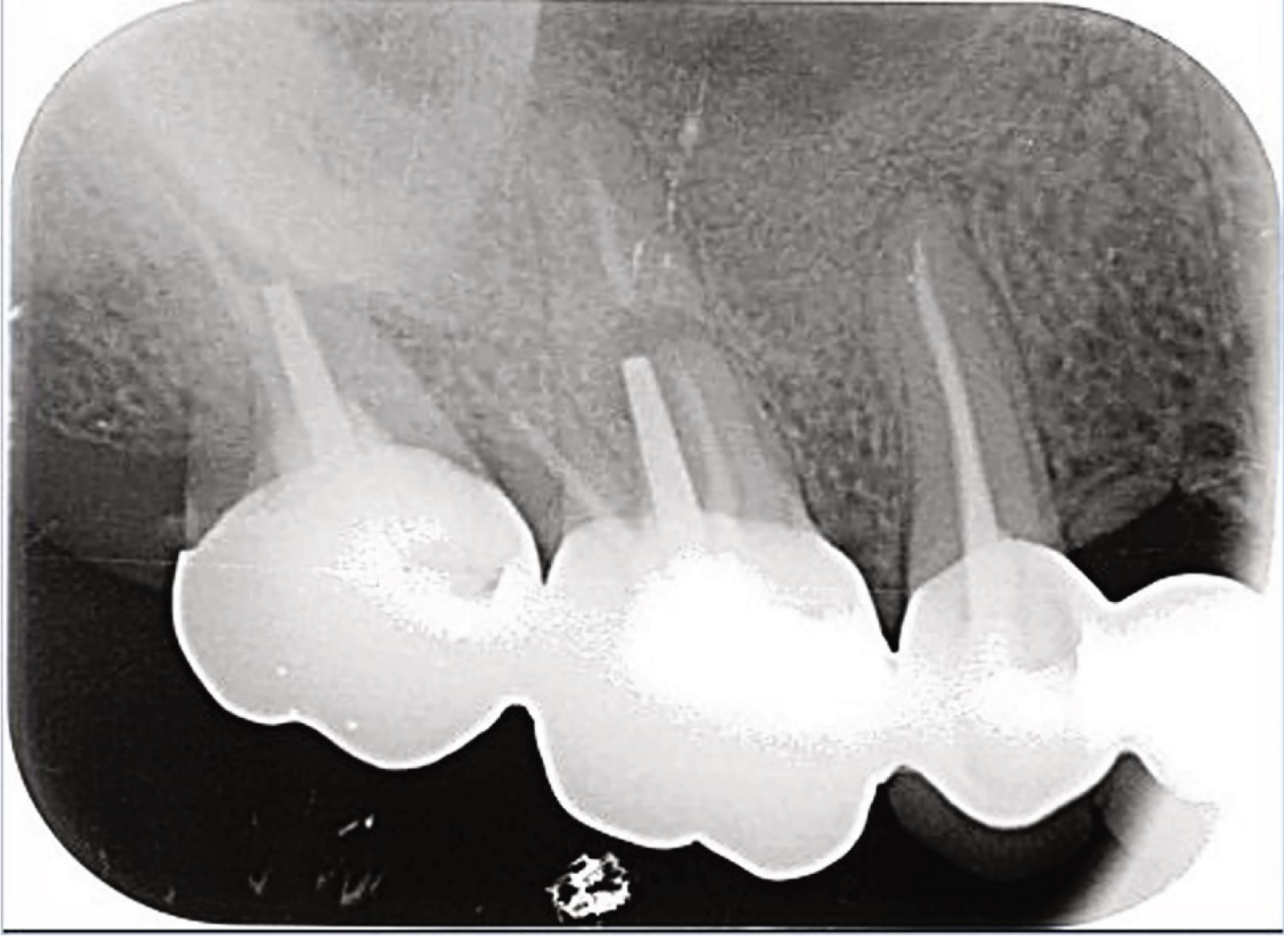
(b)

### 2.5. Dental Intervention and Outcome

Two weeks later, Tooth #16 was extracted in our hospital to achieve a complete pluridisciplinary care in the same structure. The intervention was performed under local anesthesia (articaine chlorhydrate 40 mg/mL + adrenaline 1/200,000, Septanest, Septodont, France), after separating the connectors linking the tooth to the other crowns of the bridge, with placement of a resorbable hemostatic sponge in the socket (Pangen, Urgo, France) and a single suture. The patient experienced marked clinical improvement following extraction. Subsequent prosthetic rehabilitation of the absence of Tooth #16 has then been taken care of by the patient′s usual dentist in private practice.

### 2.6. Follow‐Up

**Figure 3:**
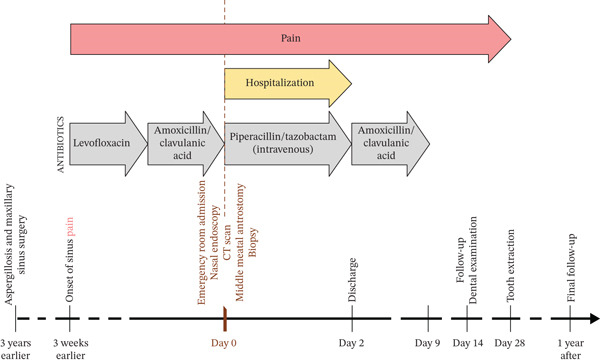
Timeline summarizing the chronological sequence of events from the onset of symptoms to the 1‐year follow‐up.

At 1 year of follow‐up, the patient reported only a transient recurrence of facial heaviness sensation in the right maxilla, which resolved spontaneously. The examination by an ORL specialist at that time showed no signs of infection or sinus abnormality. During a subsequent hospital admission for unrelated gynecological surgery later that year, the patient reported a complete resolution of maxillary symptoms. The complete chronological sequence of events concerning this case report is presented on Figure 3.

## 3. Discussion

This case underscores the therapeutic challenges of maxillary sinusitis of dental origin, particularly in this case when it presents as an ORL emergency. The patient′s initial presentation—marked by persistent facial pain, hemifacial edema, and a vestibular collection—necessitated an urgent ORL intervention. The acute nature of her symptoms, coupled with a lack of response to first‐line antibiotic therapy, raised concern for a complicated sinus infection requiring prompt surgical management.

Here, the use of iodine agent contrast in the CT scan was motivated by the symptoms, the context for emergency and the recurrent aspect of the disease. Contrast agents can enhance visualization of infection extension and differentiation between inflammatory tissue, abscess, or tumors [11, 14]. Notably, the patient′s maxillary sinusitis did not exhibit the typical drainage seen in many cases, leading to significant soft tissue infiltration and the need for emergency endonasal meatotomy. The presence of a prior Caldwell‐Luc window and a history of fungal sinusitis (aspergillosis) further complicated the clinical picture, increasing the risk of severe complications and justifying the emergency surgical approach. Despite initial resolution of symptoms following the surgical and medical management by the ORL department, the persistence and recurrence of symptoms were ultimately attributable to a periapical lesion and possible root fracture of Tooth #16. Only after dental extraction did the patient achieve lasting resolution.

The persistent nature of the patient′s symptoms necessitated careful consideration of several differential diagnoses. Given the history of aspergillosis in 2017 and also of a prior Caldwell‐Luc surgery, recurrent fungal sinusitis was a primary concern, but the treatment of fungal elements had already been proposed, and no image of calcifications or of fungus balls was visible on the CT scan [15]. This diagnosis was thus dismissed. Postoperative complications related to the previous Caldwell‐Luc procedure were not selected either, in the absence of osteonecrosis or chronic osteomyelitis on X‐rays. Refractory non‐odontogenic maxillary sinusitis was then more evident [16] with the recurrence of symptoms shortly after an apparently successful ORL management, combined with CT evidence of periapical radiolucency associated with Tooth #16.

The definitive resolution following dental extraction, with no subsequent recurrence, retrospectively confirmed that the persistent dental focus was the central determinant of symptom recurrence. In 2018, Little et al. published an interesting review on the topic of odontogenic sinusitis, in which they highlighted that a considerable amount of patients needed dental treatments plus endoscopic sinus surgery to reach a complete resolution of the disease [16]. It would thus be interesting to propose an earlier dental assessment in this type of disease, when a unilateral sinusitis fails to improve after conventional ORL‐directed therapy, to avoid diagnostic and therapeutic errancy for the patient.

The case highlights several important points:

1. The need for interdisciplinary collaboration. Successful management requires close cooperation between ORL and dental specialists. Early dental assessment should be considered immediately in cases of maxillary sinusitis unresponsive to standard therapy. This limits the time wasted in care, especially when imaging finally suggests periapical pathology.
2. The use of multiple imaging techniques. CT imaging is pivotal in identifying the dental origin of the sinusitis, and dental X‐rays also reveal subtle findings (root fracture and periapical lesion) not always apparent on clinical examination alone.
3. The need to manage each situation on a case‐by‐case basis. In our case, the presence of a previous Caldwell‐Luc window and prior aspergillosis complicated the clinical picture. Different antibiotic modalities and several stages of care were required. This can be explained if the case has been initially managed like any other sinusitis, so this highlights the need for individualized and multidisciplinary care.

In conclusion, this case illustrates that persistent maxillary sinusitis, from dental etiology, requires prompt dental assessment, even after prior sinus surgery or fungal infection. Interdisciplinary management is essential for accurate diagnosis and definitive treatment.

## Funding

This study was supported by Université Toulouse III—Paul Sabatier.

## Ethics Statement

The authors have nothing to report.

## Consent

The patient has signed an authorization to use her anonymized photographs for research and teaching purposes.

## Conflicts of Interest

The authors declare no conflicts of interest.

## Data Availability

The data that support the findings of this study are available from the corresponding author upon reasonable request.
